# Sensitivity and Acclimation of Three Canopy-Forming Seaweeds to UVB Radiation and Warming

**DOI:** 10.1371/journal.pone.0143031

**Published:** 2015-12-02

**Authors:** Xi Xiao, Thibaut de Bettignies, Ylva S. Olsen, Susana Agusti, Carlos M. Duarte, Thomas Wernberg

**Affiliations:** 1 Ocean College, Zhejiang University, Xihu District, China; 2 UWA Oceans Institute and School of Plant Biology, University of Western Australia, Crawley, WA, Australia; 3 Department of Global Change Research, IMEDEA (CSIC-UIB), Institut Mediterrani d'Estudis Avançats, Esporles, Spain; 4 Red Sea Research Center, King Abdullah University of Science and Technology, Thuwal, Saudi Arabia; University of Minnesota, UNITED STATES

## Abstract

Canopy-forming seaweeds, as primary producers and foundation species, provide key ecological services. Their responses to multiple stressors associated with climate change could therefore have important knock-on effects on the functioning of coastal ecosystems. We examined interactive effects of UVB radiation and warming on juveniles of three habitat-forming subtidal seaweeds from Western Australia–*Ecklonia radiata*, *Scytothalia dorycarpa* and *Sargassum sp*. Fronds were incubated for 14 days at 16–30°C with or without UVB radiation and growth, health status, photosynthetic performance, and light absorbance measured. Furthermore, we used empirical models from the metabolic theory of ecology to evaluate the sensitivity of these important seaweeds to ocean warming. Results indicated that responses to UVB and warming were species specific, with *Sargassum* showing highest tolerance to a broad range of temperatures. *Scytothalia* was most sensitive to elevated temperature based on the reduced maximum quantum yields of PSII; however, *Ecklonia* was most sensitive, according to the comparison of activation energy calculated from Arrhenius’ model. UVB radiation caused reduction in the growth, physiological responses and thallus health in all three species. Our findings indicate that *Scytothalia* was capable of acclimating in response to UVB and increasing its light absorption efficiency in the UV bands, probably by up-regulating synthesis of photoprotective compounds. The other two species did not acclimate over the two weeks of exposure to UVB. Overall, UVB and warming would severely inhibit the growth and photosynthesis of these canopy-forming seaweeds and decrease their coverage. Differences in the sensitivity and acclimation of major seaweed species to temperature and UVB may alter the balance between species in future seaweed communities under climate change.

## Introduction

Habitat-forming kelps and fucoids are major contributors to coastal marine food webs and provide a variety of essential ecosystem services such as provision of oxygen, shelter and nursery grounds for fauna, and removal of excess nutrients from the water column [1–3]. Habitat-forming seaweeds are highly vulnerable to environmental stressors [4–8] and are experiencing rapid decline in abundance or even local extinction along many temperate and subtropical coasts [8–11]. Therefore, understanding the responses of seaweeds to global climate change is increasingly important.

Marine organisms have developed unique thermal windows (temperature range for survival) as a result of a long history of acclimation [12]. For instance, *Ecklonia radiata* has an optimal temperature range of 18°C to 23°C for growth and reproduction [13], and a broad thermal window for occurrence of 8°C to 24°C [6]. Worldwide decline in canopy-forming brown algae (kelp and fucoid) is at least partially associated with increasing sea surface temperature, which is predicted to reach maxima exceeding their thermal windows [10,11,14]. Ocean warming can affect the physiology of canopy-forming kelps and fucoids, and lead to local extinctions and range shifts [11,15–17]. Temperature extremes, such as those experienced during marine heat waves can result in substantial changes in seaweed assemblages [8,11].

In addition to increasing mean temperatures and marine heat waves, ultraviolet radiation (UVB, 280–315 nm) has also increased in intensity due to ozone depletion, exacerbated in the Southern Hemisphere, with a strong impact on marine organisms [18,19]. UVB exposure affects photosynthesis, nitrogen metabolism, growth, and DNA in seaweeds [20], such as *Kappaphycus alvarezii* [21], *Gracilaria domingensis* [22] and *Hypnea musciformis* [23]. UVB can impact seaweeds on a celular level including increased number of cell wall-producing vesicles, thicker and denser cellular walls, as well as modification in the quantity, size and organization of chloroplasts [20,23]. Because organisms respond to a plethora of complex environmental changes at local to global scales [24], the examination of the responses of marine organisms to global change needs to adopt a multi-stressor approach, where responses to multiple (e.g. temperature and UVB) rather than single stressors, are explored [25,26].

Despite the obvious environmental relevance of increasing temperature and UVB radiation, surprisingly few studies have considered the interactive effects on seaweeds [26–31]. The response of seaweeds to thermal and UVB stress has been assessed for a limited number of species, including Rhodophyta (*Gelidium corneum*, *Gelidium pulchellum*) [32,33], Phaeophyceae (*Laminaria solidungula*, *Laminaria digitata*, *Saccharina Latissima*, *Alaria esculenta*, *Lessonia nigrescens*, *Macrocystis pyrifera and Durvillaea antarctica*) [27–30,34] and Chlorophyta (*Ulva bulbosa* and *Ulva clathrata*) [35]. Most of these studies investigated the impact of short-term incubations (e.g. from 1 hour to 5 days) to extreme UVB and temperature [29,34]. Furthermore, examinations of the response of seaweeds to thermal and UVB stress were focused largely on a variety of biochemical indicators, such as mycosporine-like amino acids, heat shock proteins, mitogen-activated protein kinases, phenolic compounds and total fatty acids [29,33,36]. In contrast, seaweed responses to these stressors in terms of their ecophysiological performance (e.g. growth rates, overall health, light absorption and photosynthesis) have rarely been assessed, although these indicators are better suited to reflect the adaption and/or acclimation of seaweeds to persistent pressures of climate change.

Habitat-forming kelps and fucoids in Western Australia have been particularly affected by combined UVB and warming. Concurrent with the high incident UVB radiation due to stratospheric ozone depletion in the Southern Hemisphere [18], and particuarly clear waters allowing deep (>10m) penetration of UVB radiation [37], the region has experienced well above global average warming over the past five decades [38], punctuated by an intense marine heat wave in early 2011. The heat wave led to a poleward regression of the northern (warm) limit of temperate kelp and fucoid habitats [8,11], and it is possible that the documented thermal vulnerability of these communities may have been exacerbated by the elevated incident UVB radiation. Yet, the response of habitat forming kelps and fucoids to the interaction of UVB and thermal stressors has not yet been evaluated in Western Australia or most other places in the Southern Hemisphere.

The west coast of Australia is a global hotspot of biodiversity and endemism, and a transition zone between tropical and temperate biota [24]. Seaweeds of the species *Ecklonia radiata*, *Scytothalia dorycarpa* and *Sargassum sp*. (hereinafter referred to as *Ecklonia*, *Scytothalia* and *Sargassum*) play a key role as foundation species in this global diversity hotspot [24]. *Ecklonia radiata* is a small kelp (1–2 m, order *Laminariales*) often dominating the seaweed flora along the temperate Australian coastline [24]. As one of the most prominent habitat-forming species across 8000 km of temperate rocky reefs [39], it forms dense and highly productivity kelp beds [40, 48]. *Scytothalia dorycarpa* is a perennial fucoid (~ 1 m, order *Fucales*) endemic to southern Australia [11] and often a co-dominant canopy-former with *Ecklonia* [41]. *Sargassum sp*. (most likely *Sargassum fallax*, 0.3–1.5 m, order *Fucales*) is also one of the most abundant large brown algae, and often form dense patches in subtidal and lower intertidal zones [41]. In contrast to *Ecklonia* and *Scytothalia*, *Sargassum* is most abundant along tropical and subtropical coasts [5] but also occurs in temperate regions [41]. These three seaweed species contribute substantially to rocky shore ecosystems in temperate and subtropical Australia.

The present study is an effort to understand how ecophysiologically resistant these key habitat-forming seaweeds are to the concurrent stressors of elevated UVB radiation and temperature. Understanding the specific sensitivities of these habitat-forming seaweeds will add new insights into the ecophysiological acclimation to concurrent components of global change in seaweed forests, which form the basis of many temperate reef ecosystems. More specifically, this knowledge will inform likely changes in the relative abundances of these species, which have been predicted to change in the future [41]. In this study, we experimentally investigated the importance of UVB radiation and warming in regulating the performance of juvenile *Ecklonia radiata*, *Scytothalia dorycarpa* and *Sargassum sp*. Fronds were exposed to a range of temperatures in the presence or absence of UVB radiation and assessed for growth rate, health index, photosynthetic yield and tissue light absorption. Furthermore, we used empirical models from the metabolic theory of ecology to evaluate the sensitivity of these important seaweed species to ocean warming.

## Materials and Methods

### Algal material, location and pretreatment

Juveniles of *Ecklonia radiata*, *Scytothalia dorycarpa* and *Sargassum sp*. (most likely *S*. *fallax*) were collected by SCUBA divers at Centaur Reef (S 31°51', E115°42'), Marmion (25 km north of Perth, Western Australia). These species are common and this study did not involve any protected or endangered species. The average lengths of three seaweeds were 18, 8, and 17 cm, respectively. Over 100 individuals of each seaweed species were collected from a subtidal rocky reef between 8–12 m deep during austral mid-winter (August 2014). The seaweeds were kept in dark plastic containers with aerated seawater at ambient seawater temperature (about 17–18°C) while transported to the laboratory. After carefully removing epiphytes, all thalli were attached to individual pebbles and placed in a 100 L aquarium for initial acclimation under continuous air bubbling and circulation of filtered seawater (10°C, low light) for 5 days before the experiments were initiated, to minimize any stress following collection and translocation to the laboratory. The seaweeds were kept under gradually increasing temperatures for 1–2 days, until reaching the temperatures for the experimental incubations.

### Experimental design

Experiments were conducted under a broad temperature range from 16°C to 30°C with 1 to 2°C intervals, capturing and exceeding the entire current temperature range where the three seaweed species are found in southern and western Australia [13,41]. Healthy (brown, no epiphytes, and no visible damage) and equally sized individuals of *Ecklonia*, *Scytothalia* and *Sargassum* were selected from the acclimation aquarium. Two thalli of each species were placed in each of twenty 30-L aquaria. Temperature treatments were established at 16, 18, 19, 21, 22, 24, 25, 27, 28 and 30°C with two aquaria per temperature. Electronic heaters (Aqua One 55W, Aqua Pacific, Southampton, UK; Aqua Heater 100W, WN, Foshan, China) and chillers (Teco TC15, Teco S.r.l., Ravenna, Italy) were used to control the temperatures and temperature loggers (Hobo-UA-002-64, Onset, Massachusetts, USA) were set up to continuously record the temperature inside each aquarium.

The aquaria were illuminated using PAR lamps (Daylight, 110–150 μmol m^-2^ s^-1^, natural photo period of 8L: 16D; Philips, Netherlands). To examine the effect of UVB, half of the aquaria were illuminated using a combination of UVB lamps (VL-8.M 312nm Lamp, Vilber Lourmat, France) and PAR lamps. This resulted in one aquarium for each temperature and light condition (UVB+ or UVB-). To simulate natural conditions found in southwestern Australia [37], the UVB radiation at canopy height was in the range of 0.05–0.07 mW cm^-2^, with an exposure time of 5 hours day^-1^. UVB radiation was measured using a radiometer (PMA2100, Solar Light Co., Inc, Glenside, USA). During the experiments, all aquaria were aerated and supplied with circulating filtered seawater. Nutrients were supplied into the seawater as PES medium (Provasoli´s Enriched Seawater medium) twice per week.

### Growth rates

At the beginning of the experiment algal fronds were marked by either punching a small hole (diameter 1.5 mm; for *Ecklonia* and *Scytothalia*) or attaching a small cable tie (for *Sargassum*), around 3 cm from the top of stipe. The distance from the top of the frond to the mark, and the wet weight of thallus, were measured for each individual. After exposure to the experimental treatments for 2 weeks, distances and wet weights of the seaweeds were re-measured, and net biomass accumulation (NBA, % day^-1^) and linear growth rate (LGR, % day^-1^) were estimated as:

$$NBA=100(lnW_{t}\;-\;lnW_{0})/t$$ (1)

$$LGR=100(lnD_{t}\;-\;lnD_{0})/t$$ (2)

with *W*_*0*_ (or *D*_*0*_) = initial wet weight (or distance); *W*_*t*_ (or *D*_*t*_) = wet weight (or distance) at time t since the beginning of the experiment.

### Optimum quantum yield of PSII

Optimum quantum yield (F_v_/F_m_) of photosystem II (PSII) was determined on days 0, 2, 7, 10 and 14 for all individuals by pulse amplitude modulated (PAM) fluorometry (MINI-PAM-II, Waltz, Effeltrich, Germany), as an indicator of PSII integrity and corresponding photosynthetic efficiency. Prior to measurements fronds were dark adapted for 15 min to open all antennae pigments, taking the tissue back to a baseline reflecting the physiological capacity of the tissue without the influence of ambient conditions.

### Health status

The ‘health status’ of the seaweeds was assessed visually on days 0, 2, 7, 10 and 14 as the percentage of ‘unhealthy’ tissue [42,43].

### Spectral *in vivo* light absorption properties

Light absorption was measured *in vivo* on the thalli of 6 juveniles of each species before the experiment to generate a baseline. To assess the effect of UVB and temperature on spectral light absorption properties, measurements were carried out at the end of the experiment (Day 14) on healthy tissue sections from the 16°C and 30°C treatments. Light absorption was measured every nanometer between 280 nm to 800 nm using the opal glass technique [44] with a double beam spectrophotometer (Cary 3, Agilent Tech., Santa Clara, USA). The change from tungsten to deuterium lamp was set at 350 nm. A UV transparent fused silica ground glass diffuser (Edmund Optics Inc., Barrington, USA) was used to ensure the diffusion of light within both the UV and visible spectral bands. Absorbance was recorded and corrected by subtracting the value at 725 nm from all spectral values to exclude residual scattering. The corrected absorbance values were then transformed to absorptance following the equation:

$$Absorptance=1-10^{-abs}$$ (3)

where abs is the measured absorbance at the spectrophotometer [44].

### Activation energy from the Arrhenius equation

The Boltzmann–Arrhenius model for chemical reaction kinetics was used to predict the metabolic rate, from the perspective of metabolic theory of ecology [45]. Temperature governs metabolism through the effects on biochemical reaction rates, following the Arrhenius equation:

$$R=\;R_{0}e^{-E/kT}$$ (4)

where R is biological rate (in this case the health status), *k* is Boltzmann’s constant, T is thermodynamic temperature and E is activation energy. We estimated the activation energy for plants with or without UVB after one week and two weeks of incubation by plotting ln(R) as a function of 1/kT, where R was the health status of the algae, ln(R) was the natural logarithm of the percentage of unhealthy plants and T was the range of experimental temperatures in degrees K.

### Data analysis

All seaweed treatment bioassays and measurements were conducted in duplicate, i.e. on two fronds for each species in each aquarium. Ordinary Least Square (OLS) linear regression was used to model seaweed growth rates as a function of temperature, UVB radiation and their interaction. The activation energy was calculated from the slope of the Arrhenius model fitted using linear regression of ln (% unhealthy) as a function of 1/kT. Significance analysis between control and treated samples (paired samples *t*-test) was carried out with R (version 3.1.2). *p* < 0.05 was considered to be statistically significant. All data are available from the Dryad repository: http://datadryad.org/resource/doi:10.5061/dryad.gt6ks.

## Results

### Growth and biomass accumulation

**Fig 1:**
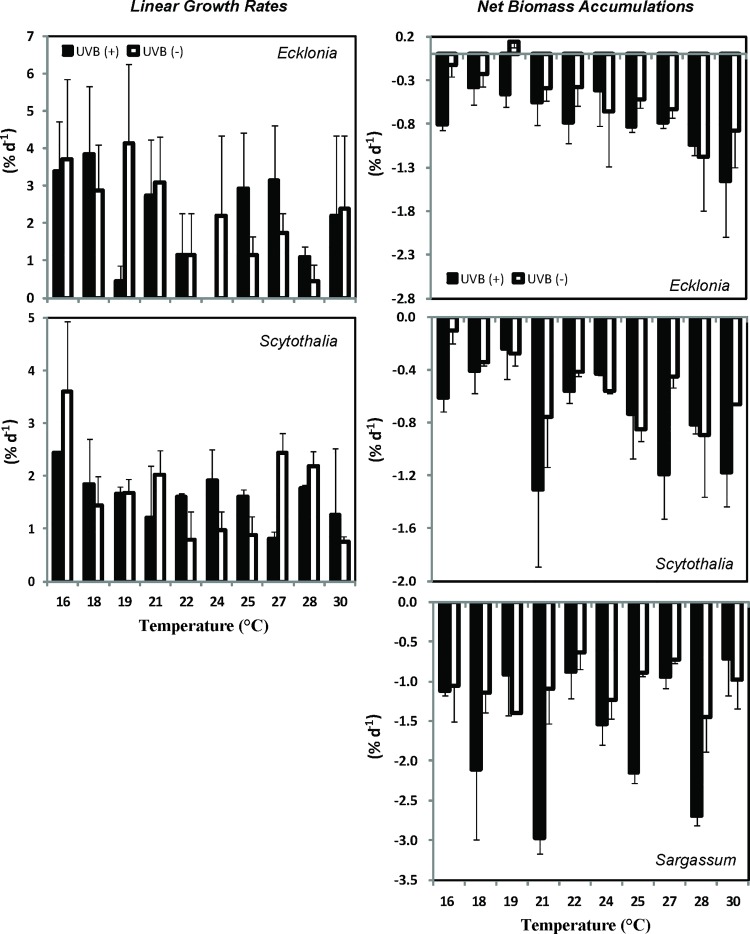
Linear growth rate (LGR) and net biomass accumulation (NBA) of *Ecklonia*, *Scytothalia* and *Sargassum* cultivated at 16 to 30°C, with (UVB (+), filled bars) or without (UVB (-), open bars) exposure to ultraviolet B radiation. Values shown are means ± standard errors.

Linear growth rates (LGR) of *Ecklonia* and *Scytothalia* showed no significant differences among temperature treatments after 14 days of incubation (Fig 1, Table 1), however, there was a tendency for the LGR of both species to decline under warmer conditions (Fig 1). *Sargassum* grew less than 1 mm (LGR) in all controls and treatments and the results are therefore not shown. In general, the net biomass accumulation (NBA) was negative (i.e. biomass was lost) (Fig 1). Elevated temperature significantly increased biomass loss of *Ecklonia* (p < 0.05) (Fig 1; Table 1). UVB-exposed juveniles showed greater loss of biomass than those not exposed (Fig 1, Table 1), although the effect of UVB on LGR was not significant (Table 1). Variations in the measurements and the relatively slow growth rate of seaweeds (i.e. *Sargassum*) may have contributed to the lack of significant effects.

**Table 1:** Statistics from OLS linear regression on the growth rates of *Ecklonia*, *Scytothalia* and *Sargassum*, as a function of temperature, UVB radiation and their interaction. NBA: net biomass accumulation rate (% day^-1^); LGR: linear growth rate (% day^-1^).

	Seaweed Species	Factor			p value		
		TEMP	UVB	TEMP*UVB	TEMP	UVB	TEMP*UVB
LGR	*Ecklonia*	-0.178	-2.869	0.116	0.052	0.322	0.348
	*Scytothalia*	-0.080	-0.597	0.023	0.124	0.720	0.746
NBA	*Ecklonia*	-0.072	-0.799	0.023	**0.001**	0.162	0.333
	*Scytothalia*	-0.040	-0.112	-0.005	0.054	0.863	0.871
	*Sargassum*	0.008	-0.472	-0.003	0.870	0.765	0.964

### PAM fluorometry

**Fig 2:**
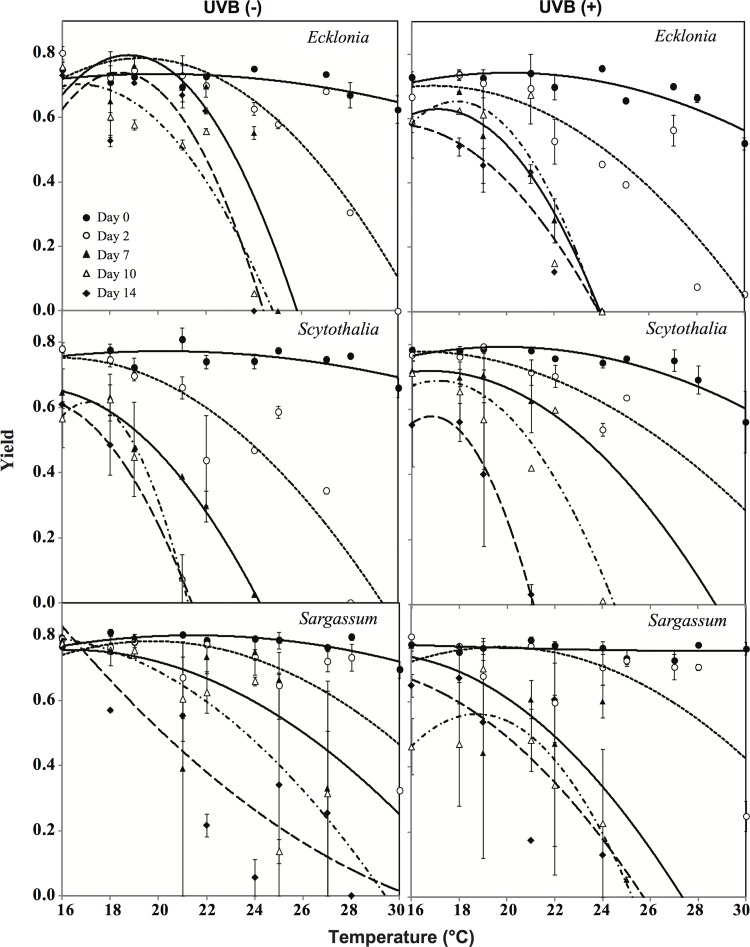
Maximum quantum yield values (mean ± standard error) measured on dark-adapted *Ecklonia*, *Scytothalia* and *Sargassum*, after 0, 2, 7, 10 and 14 days of cultivation at temperatures from 16 to 30°C, left: no UVB radiation; right: exposed to UVB radiation.

**Fig 3:**
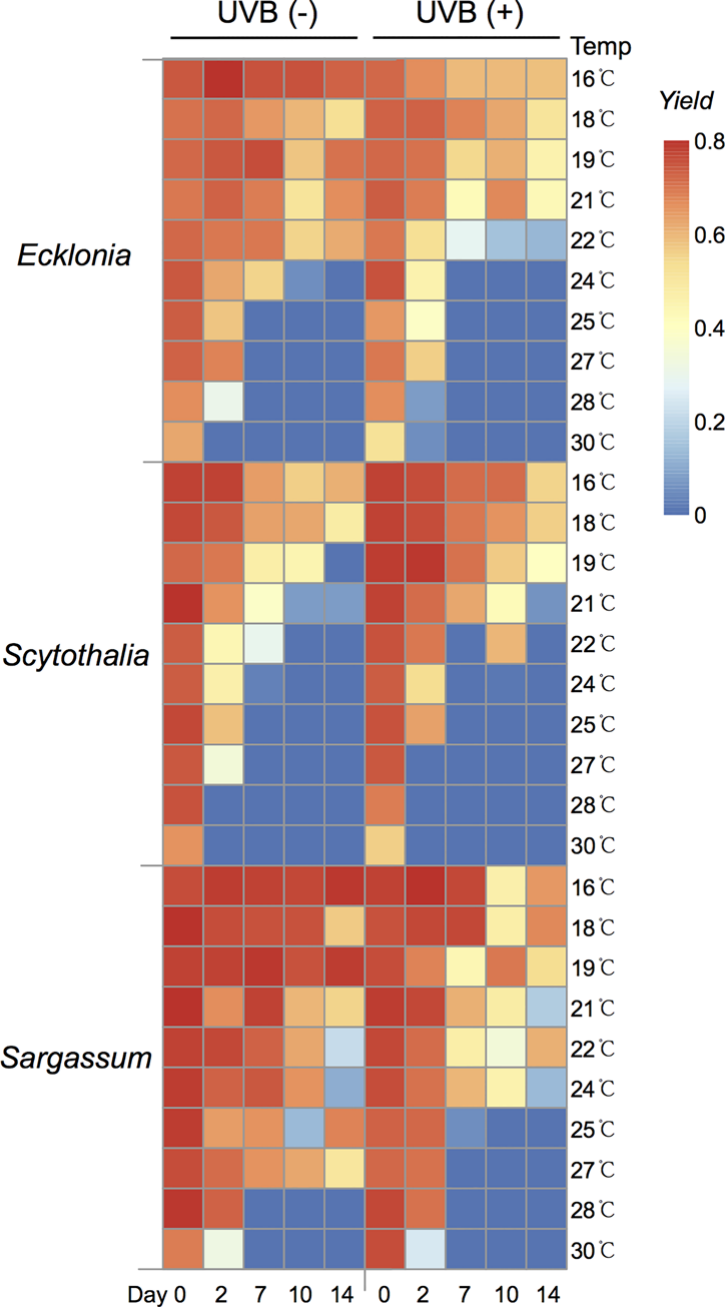
Maximum quantum yield of dark adapted *Ecklonia*, *Scytothalia* and *Sargassum* after 0, 2, 7, 10 and 14 days of cultivation at temperatures from 16 to 30°C, left: no UVB radiation; right: under UVB radiation.

The maximum quantum yields measured before the experiment were similar for *Ecklonia*, *Scytothalia* and *Sargassum*, (0.75, 0.78 and 0.76, respectively; Fig 2). Maximum quantum yields for seaweeds grown under control conditions (16°C and without UVB) remained at this level for all three species for the duration of the experiment, with only a slight decrease for *Scytothalia* towards the end (Fig 3). Maximum quantum yield declined with increasing temperature in all species and approached zero in all three species after 14 days of incubation at the highest temperatures (Fig 3). Individuals of *Scytothalia* incubated above 19°C showed no photosynthetic activity at day 14. In contrast, *Sargassum* was relatively resistant to elevated temperature (Fig 3), with maximum quantum yield of 0.51 observed under the 27°C treatment without UVB at the end of experiment (Fig 2).

UVB caused a further reduction in quantum yield for all three species (Fig 2 and Fig 3). At day 14, UVB resulted in an average 35%, 3% and 13% reduction across all temperatures in Fv/Fm for *Ecklonia*, *Scytothalia* and *Sargassum*, respectively. Interestingly, the maximum quantum yields for *Scytothalia* at days 10 and 14 were higher in the UVB-exposed individuals than those unexposed (Fig 3).

### Temperature dependency of macroalgal health

**Fig 4:**
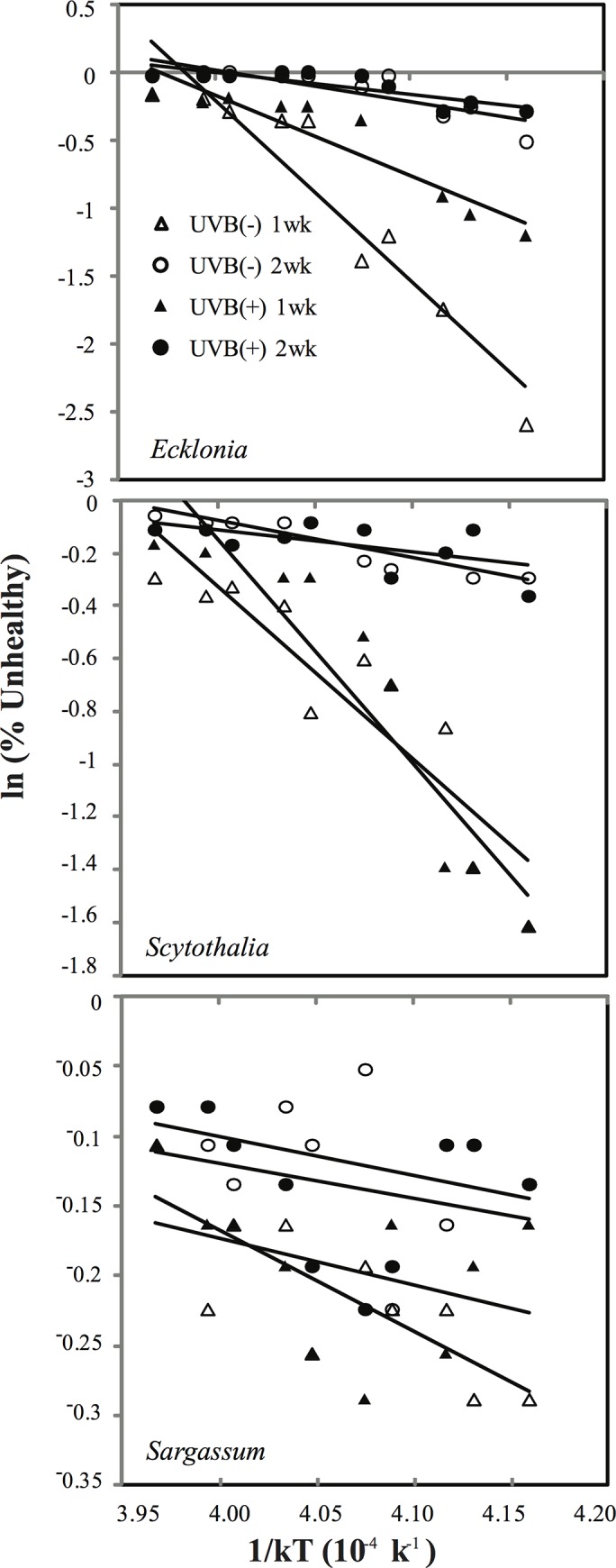
The natural logarithm of the proportion of unhealthy fronds of *Ecklonia*, *Scytothalia* and *Sargassum* incubated at 16 to 30°C with (black) or without (white) UVB radiation. Incubation temperatures are represented as 1/kT, where T is thermodynamic temperature and k = 8.31 J/mol. The % of unhealthy fronds was assessed after 7 (triangles) and 14 days (circles). The slope of each linear regression is equal to the activation energy (E) and is given in Table 2.

The deterioration in health status of the three seaweed species with warming was consistent with the Arrhenius model, based on OLS regressions (R^2^ > 0.5, p < 0.05 for the regressions), allowing calculation of the corresponding activation energies reflecting the temperature dependency of seaweed health (Fig 4, Table 2). For the two time-periods and two UVB treatments, activation energies (i.e. thermal sensitivity) of the seaweeds followed the sequence: *Ecklonia* > *Scytothalia* > *Sargassum* (Table 2). The activation energy ranged from 0.16 eV (p < 0.001) to 1.36 eV (p < 0.01) for *Ecklonia*, from 0.15 eV (p < 0.001) to 0.88 eV (p < 0.001) for *Scytothalia*, and from 0.03 eV (p < 0.001) to 0.08 eV (p < 0.01) for *Sargassum* (Table 2). Hence, *Sargassum* was most resistant to elevated temperature while *Ecklonia* was most vulnerable, based on the thermal responses of plant health.

**Table 2:** Activation energy based on the Arrhenius model using the OLS linear regression of ln (% Unhealthy) as a function of 1/kT.

Species	UVB	Day	Energy	R_0_	R^2^	p value
*Ecklonia*	(-)	7	1.36	52.47	0.896	**0.0001**
	(-)	14	0.24	9.35	0.707	**0.0023**
	(+)	7	0.61	23.39	0.869	**0.0002**
	(+)	14	0.16	6.35	0.704	**0.0024**
*Scytothalia*	(-)	7	0.68	25.78	0.819	**0.0003**
	(-)	14	0.15	5.55	0.827	**0.0003**
	(+)	7	0.88	33.81	0.867	**0.0001**
	(+)	14	0.09	3.31	0.347	0.0730
*Sargassum*	(-)	7	0.08	2.75	0.626	**0.0064**
	(-)	14	0.03	1.03	0.133	**0.0003**
	(+)	7	0.03	1.16	0.143	0.2822
	(+)	14	0.03	0.89	0.096	0.3829

Exposure to UVB radiation appeared to reduce the thermal sensitivity of seaweed health, as reflected in their activation energy, compared to those without UVB exposure (Fig 4, Table 2). For example, the activation energy of *Ecklonia* was 1.36 eV in the absence of UVB radiation, but decreased to 0.61 eV when the seaweeds were grown under UVB radiation at day 7. These differences in activation energies imply that UVB-exposed seaweeds are less sensitive to temperature than unexposed algae. The only exception was for *Scytothalia* at day 7, when the activation energy showed a 29.4% increase (Table 2). Again, this may be caused by the rapid acclimation of *Scytothalia* to UVB, as supported by the relatively high values of maximum quantum yield of PSII of UVB exposed individuals found at days 10 and 14 (Fig 3).

The activation energy decreased progressively along the experiment for all three seaweeds, with an average of 64.4% reduction, suggesting an increase in the resistance of seaweeds to elevated temperature over time, probably because the juveniles were gradually acclimated to the environmental conditions. At day 14, activation energy measured in *Ecklonia*, *Scytothalia* and *Sargassum* were 33.3%, 40.0% and 0.0% lower in UVB treatments, indicative of differences in thermal resistance between species.

### *In vivo* light absorption spectra

**Fig 5:**
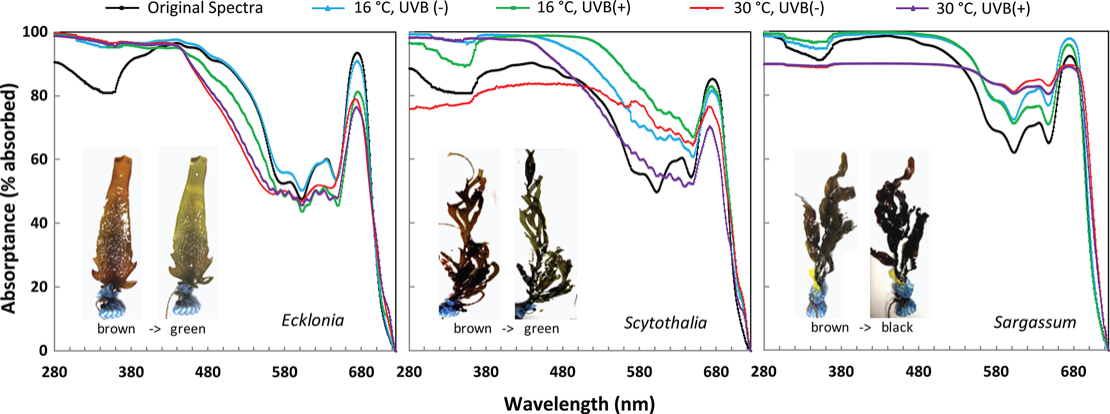
Light absorption spectra of *Ecklonia*, *Scytothalia* and *Sargassum* before incubation (original spectra) and after 14 days of cultivation at 16°C and 30°C, with (UVB (+)) or without (UVB (-)) UVB radiation. The spectra are averaged for all individuals (n = 2~6) in each treatment. Photos illustrate transitions from “healthy” to “unhealthy” status: discoloration from brown to green for *Ecklonia*, *Scytothalia*; and becoming darker from brown to black for *Sargassum*.

The three seaweed species tested showed different spectral light absorption properties (Fig 5). *Ecklonia*, *Scytothalia* and *Sargassum* absorbed, on average, 76.2%, 73.5% and 83.9% of the incident light (average PAR absorption) (Fig 5, Table 3). *Sargassum* had significantly higher UVB light absorption ability (97.0±0.4%) in the bands from 280 to 320 nm (Table 3), compared to the other two species (87.9±3.2% and 85.2±7.6% for *Ecklonia* and *Scytothalia*, respectively). The absorption in the band corresponding to Mycosporine-like amino acids (MAAs, a group of photoprotective compounds common in macroalgae), as well as the accessory pigments fucoxanthin and Chlorophyll *c* (*Chl c*) were also highest in *Sargassum* (Table 3). Both *Ecklonia* and *Sargassum* showed a larger *in vivo* absorption by Chlorophyll *a* (*Chl a*) at 675 nm (93.5±1.3% and 92.3±3.2%, respectively) than *Scytothalia* (85.3±1.3%). However, the ratio of MAAs and fucoxanthin to *Chl a* absorption followed the sequence of *Ecklonia* < *Scytothalia < Sargassum* (Table 3).

**Table 3:** Light absorption properties of thalli for *Ecklonia*, *Scytothalia* and *Sargassum* at the beginning of the experiment and after 14 days of cultivation at 16 and 30°C, with or without exposure to UVB radiation. Data shows absorptance values for *in vivo* Chlorophyll *a* (675 nm), Chlorophyll *c* (635.5 nm), Fucoxanthin (590 nm), and maximum absorption in the MAAs peak absorption range (309–360 nm); the ratio of MAAs and Fucoxanthin to Chlorophyll *a*; and the averaged values in the wavelength-bands of UVB (280–320 nm) and PAR (400–700 nm).

Species	*Treatments*		*Absorptance (% absorbed)*						*Light absorption (%)*	
	Temperature (°C)	UVB^a^	*Chl a*	MAAs	Fucoxanthin	MAAs/*Chl a*	Fuc./*Chl a*	*Chl c*	UVB	PAR
	Original absorptance^b^		93.5 ±1.3	86.4 ±2.6	52.6± 3.1	0.92	0.56	60.1± 3.2	87.9 ±3.2	76.2 ±2.1
	16	(-)	91.0± 3.1	97.5 ±0.7	55.4 ±4.4	1.07	0.61	59.7 ±5.4	98.4 ±0.4	77.0 ±3.2
*Ecklonia*	16	(+)	81.3 ±7.5	98.4 ±0.3	48.1 ±7.4	1.21	0.59	50.3 ±8.7	99.0 ±0.2	70.2± 6.5
	30	(-)	78.3 ±8.8	98.7 ±9.0	48.7 ±0.5	1.26	0.62	51.3 ±4.5	99.2 ±12.4	67.8± 3.0
	30	(+)	76.4 ±5.0	98.1 ±0.2	48.3 ±6.8	1.28	0.63	48.5 ±6.5	98.4 ±0.4	67.9 ±5.6
	Original absorptance^b^		85.3 ±7.8	82.9 ±6.4	54.6± 2.0	0.97	0.64	60.4 ±3.0	85.2 ±7.6	73.5 ±4.3
	16	(-)	81.6 ±8.0	98.7 ±0.6	69.5 ±17.5	1.21	0.85	64.8 ±18.6	98.9 ±0.4	81.8 ±11.0
*Scytothalia*	16	(+)	82.7^c^	94.5^c^	80.0^c^	1.14	0.97	71.1^c^	95.3^c^	86.9^c^
	30	(-)	75.4^c^	78.2^c^	74.4^c^	1.04	0.99	66.3^c^	76.2^c^	76.4^c^
	30	(+)	69.2^c^	98.3^c^	58.3^c^	1.42	0.84	52.3^c^	98.2^c^	72.8^c^
	Original absorptance^b^		92.3 ±3.2	95.0 ±0.5	67.4 ±3.3	1.03	0.73	71.2 ±3.1	97.0 ±0.4	83.9 ±2.2
	16	(-)	97.8 ±3.4	96.8± 6.3	77.4 ±8.9	0.99	0.79	82.2 ±9.5	97.9 ±7.7	89.9 ±5.4
*Sargassum*	16	(+)	95.6 ±5.3	98.1 ±0.2	76.3 ±8.2	1.03	0.80	76.5 ±9.0	98.8 ±0.1	88.3± 6.0
	30	(-)	89.5 ±11.4	89.2 ±1.9	83.2 ±5.6	1.00	0.93	84.9± 8.1	89.5 ±2.0	86.9± 4.8
	30	(+)	88.9 ±6.2	89.6 ±2.2	82.7 ±7.5	1.01	0.93	82.8± 8.6	89.7 ±2.5	86.3 ±5.3

^a^ UVB: (+) represents seaweeds were exposed to ultraviolet B radiation; (-) represents seaweeds were not being exposed to UVB radiation.

^b^ Absorptance of seaweeds at the beginning of experiment (Day 0).

^c^ For the three treatments of 16°C with UVB exposure and 30°C with/without UVB, samples of health section could be found from only one replicate of seaweeds (no SE shown).

We observed differences in light absorption among treatments after 14 days of exposure to elevated temperature and UVB radiation (Fig 5). Overall light absorption decreased under thermal and UV stress, indicating a decrease in pigmentation, which was also observed as discoloration in the seaweed and reflected in their health status; from brown to green for *Ecklonia* and *Scytothalia*; and from brown to black for *Sargassum* (Fig 5). These observations coincided with changes in the spectra for seaweeds. Temperature induced greater changes on the light absorption properties of all three macroalgal species than UVB (Fig 5, table 3). For instance, UVB did not affect light absorption of *Chl a* in *Ecklonia*, but there was a 17.3% reduction of light absorption of *Chl a* in *Ecklonia* at 30°C compared to 16°C (Fig 5, Table 3).

The absorption spectra and pigment composition of all three species changed after 14 days of experimental incubation. Decrease of pigments were observed in all three species under warming, i.e. *Chl a* for *Ecklonia*, both *Chl a* and fucoxanthin for *Scytothalia*, and in the spectral band corresponding to MAAs for *Sargassum* (Fig 5, Table 3). The efficiency of light harvesting by seaweeds was reduced under stress (Table 3). For instance, warming increased the ratio of MAAs/*Chl a* in *Ecklonia* (37.0%) and *Scytothalia* (24.7%), and the ratio of Fucoxanthin/*Chl a* in *Scytothalia* (54.7%) and *Sargassum* (27.6%) (Table 3). Elevated temperature caused significant changes in the PAR range of light absorption for *Ecklonia* and in the UVB range for *Sargassum* (Table 3). UVB caused a slight increase in the ratio of MAAs/*Chl a* for all three species (Table 3).

## Discussion

This study found differences in the sensitivity and acclimation to warming and UVB radiation of major co-occurring seaweeds. These observations illustrate physiological processes that might underpin future changes in the relative species distribution. All three species were negatively affected by warming, however *Sargassum* showed the broadest temperature tolerance. These results are consistent with previous studies that a sustained increase in temperature will reduce growth and productivity of several brown seaweeds [11,46,47]. As a strong driver of species distribution, temperature directly affects the survival, growth and recruitment of seaweeds [46].

UVB radiation was found to cause reduction in the growth and physiological responses in all three species, as well as increase in the abundance of unhealthy thalli. Only *Scytothalia* increased its light absorption efficiency in the UVB bands after the 14 days. UVB radiation has been shown to cause a sharp decline in performance and increase in mortality across many marine taxa including seaweeds in both hemispheres [18,19].

Elevated temperatures showed antagonistic effects with exposure to UVB. For instance, maximum quantum yield for *Scytothalia* at day 10 and 14 were higher in the UVB-exposed individuals than those unexposed (Fig 3). One possible explanation is that the UV-induced reduction of photosynthesis efficiency in *Scytothalia* was compensated by elevated temperature (Fig 3), as it has been reported for higher plants [48–50]. Another possibility could be that *Scytothalia* acclimated in response to daily doses of UVB, which has been previously observed for other brown and red seaweeds [31,51].

This study demonstrated that warming could alleviate the negative effect of UVB on growth. This result was unexpected, highlighting our limited understanding of the interactions between warming and UVB radiation and other global change factors [48]. Yet, our findings are consistent with the reports that increased temperature promotes photo-damage repair in plants, including cucumber, maize and sunflower [48–50]. In the green seaweeds *Ulva bulbosa* and *Ulva clathrata*, low temperatures have also been suggested to enhance UV-induced photosynthetic stress [35]. This finding can explain the apparent antagonistic effects of temperature and UVB on seaweed performance. In contrast, several brown seaweeds (i.e. *Fucus spiralis*, *F*. *vesiculosus* and *F*. *serratus*) were found to be highly vulnerable to UVB radiation under high temperature [52].

UVB radiation is believed to inhibit seaweed growth by damaging key enzymes involved in energy generation (i.e. photosynthesis) or consumption (i.e. respiration, pigment synthesis) processes [53]. Temperature also affects enzyme activity across biochemical processes [45]. Thus, the effect of the interaction of UVB and temperature in seaweeds depends on the combined effect of both factors. Thermal and UVB stress often co-occur because of seasonal changes, as seaweeds are exposed to both increased temperature and UV radiation during emersion [34]. High solar stress can lead to the accumulation of MAA, which plays the role of a primary shielding barrier against UVB radiation [18,36].

Absorbance of UV irradiance was greater than absorbance of photosynthetically active radiation in all three seaweeds, as observed in most algal taxa [54,55]. UVB radiation may be pre-captured by UV absorbing compounds [55], such as MAAs [18,36]. The ratio of MAAs/*Chl a* increased over time (Table 3, Fig 5). The results therefore suggested that warming had a more negative impact on photosynthesis than UVB through thermal damage to photosynthetic pigments, such as Chl a and Chl c. The different changes of absorption spectra for the three seaweeds suggested different acclimation mechanisms in response to UVB radiation and warming. *Ecklonia* and *Scytothalia* became greener under stress compared to their initial healthy brown color, which is consistent with the decrease of accessory pigments reflected by the *in vivo* light absorption spectra (Fig 5). Fucoxanthin in *Sargassum* was up-regulated as the temperature increased, which would enhance the light absorption and make the seaweed darker in color.

We used the Arrhenius model to examine the responses of seaweeds to warming, allowing comparisons between different species [45]. Differences in activation energy usually reflect different selection pressures for physiological and ecological traits of microbes, plants and animals [45]. Hence, the different activation energies derived for the three species tested here suggest they may be differentially impacted with future climate change.

In a warmer ocean *Sargassum* may have an advantage as it tolerated a broader range of temperatures. In contrast, *Scytothalia* performed better under increased UVB levels, where it could be the best competitor, because of its fast acclimation ability to UVB radiation. Differences among these three canopy forming algae in their responses to warming and UVB may have implications for their relative abilities to adapt and compete. Moreover, our results also suggested that elevated temperature and UVB could limit the growth and photosynthesis of the canopy-forming seaweeds and affect their cover, as it has been observed in field surveys across temperature gradients in this region [8].

Current research on the impact of environmental pressures on seaweeds often measures responses in DNA, physiological and biochemical parameters during short-term experiments [29,34,36], however these responses do not always translate into a change in growth or survival [31]. To better elucidate ecological effects of climate change on seaweeds, future studies should investigate more integrative and ecologically relevant processes reflecting growth, survival and reproduction, in addition to the physiological and biochemical responses commonly used. Moreover, the magnitude of the responses measured here declined over the duration of the experiment, suggesting an acclimation capacity. Short-term experiments may therefore overestimate the impacts. UVB and high temperature appeared to have antagonistic effects, further calling for the need for multi-stressor approaches [25,26] to assess responses of marine organisms to multiple concurrent stresses.

## Supporting Information

S1 Fig Health status of *Ecklonia*, *Scytothalia* and *Sargassum* incubated at temperatures from 16 to 30°C, with (black) or without (white) UVB radiation (triangles: after 1 week; circles: after 2 weeks). (DOCX)
