# Supplementary material for: Sensitivity and Acclimation of Three Canopy-Forming Seaweeds to UVB Radiation and Warming
Source: PLoS One. 2015 Dec 2;10(12):e0143031. doi: 10.1371/journal.pone.0143031 (PMC4668109; doi:10.1371/journal.pone.0143031)

**S1.** Health status of *Ecklonia*, *Scytothalia* and *Sargassum* cultivated at temperatures from 16 to 30 °C, with (black) or without (white) UVB radiation (triangles: after 1 week; circles: after 2 weeks).


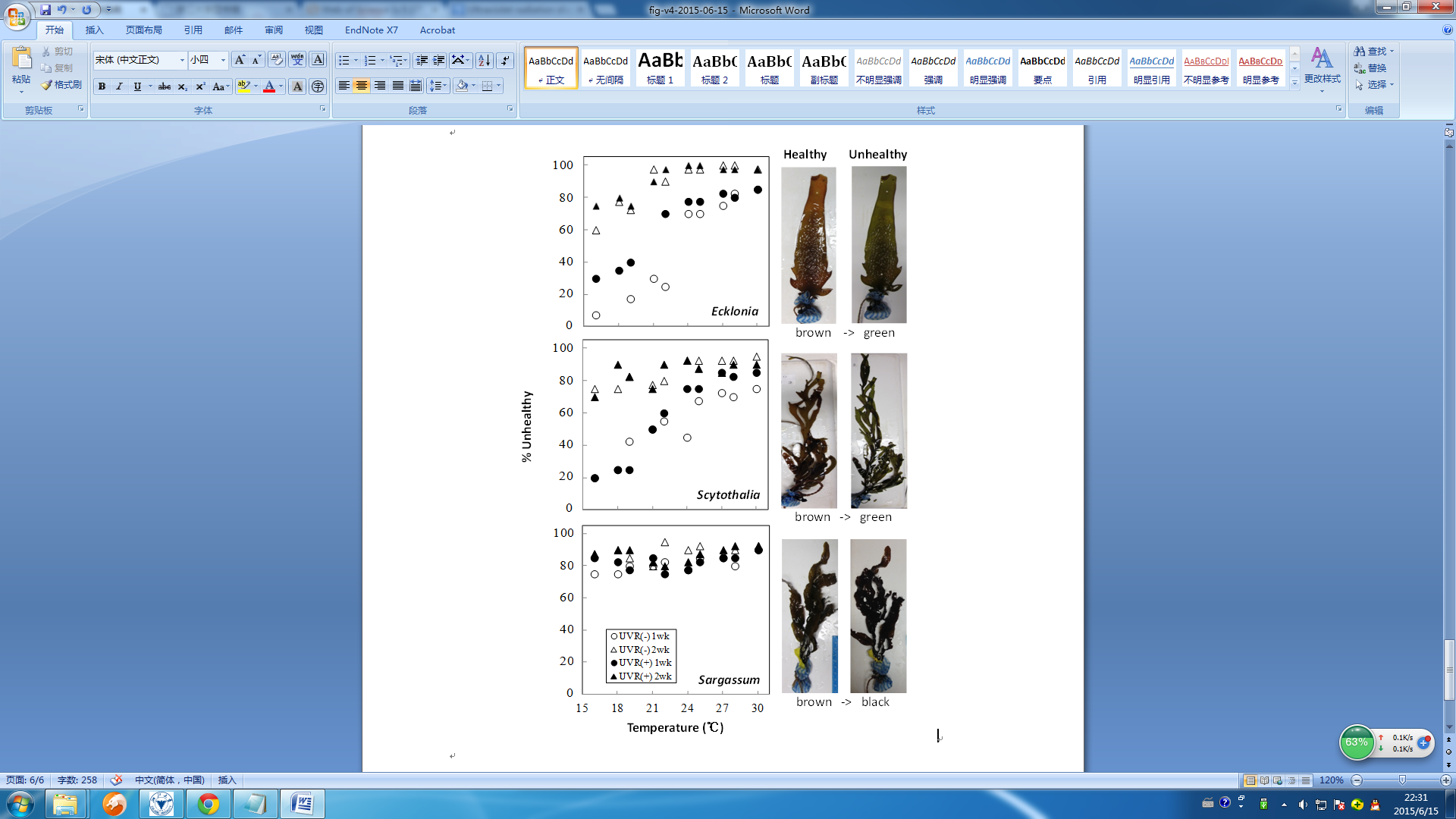

Supplement: S1 Fig — (DOCX) [file pone.0143031.s001.docx]
